# Cysteine protease enhances plant-mediated bollworm RNA interference

**DOI:** 10.1007/s11103-013-0030-7

**Published:** 2013-03-04

**Authors:** Ying-Bo Mao, Xue-Yi Xue, Xiao-Yuan Tao, Chang-Qing Yang, Ling-Jian Wang, Xiao-Ya Chen

**Affiliations:** National Key Laboratory of Plant Molecular Genetics, National Plant Gene Research Center, Institute of Plant Physiology and Ecology, Shanghai Institutes for Biological Sciences, Chinese Academy of Sciences, 300 Fenglin Road, Shanghai, 200032 People’s Republic of China

**Keywords:** Cysteine proteases, Peritrophic matrix, *Helicoverpa armigera*, Gossypol, RNAi, Insect control

## Abstract

Oral ingestion of plant-expressed double stranded RNA (dsRNA) triggers target gene suppression in insect. An important step of this process is the transmission of dsRNA from plant to midgut cells. Insect peritrophic matrix (PM) presents a barrier that prevents large molecules from entering midgut cells. Here, we show that uptake of plant cysteine proteases, such as GhCP1 from cotton (*Gossypium hirsutum*) and AtCP2 from *Arabidopsis*, by cotton bollworm (*Helicoverpa armigera*) larvae resulted in attenuating the PM. When GhCP1 or AtCP2 pre-fed larvae were transferred to gossypol-containing diet, the bollworm accumulated higher content of gossypol in midgut. Larvae previously ingested GhCP1 or AtCP2 were more susceptible to infection by *Dendrolimus punctatus* cytoplasmic polyhedrosis virus (DpCPV), a dsRNA virus. Furthermore, the pre-fed larvae exhibited enhanced RNAi effects after ingestion of the dsRNA-expressing plant. The bollworm P450 gene *CYP6AE14* is involved in the larval tolerance to gossypol; cotton plants producing dsRNA of *CYP6AE14* (*dsCYP6AE14*) were more resistant to bollworm feeding (Mao et al. in Transgenic Res 20:665–673, 2011). We found that cotton plants harboring both *35S:dsCYP6AE14* and *35S:GhCP1* were better protected from bollworm than either of the single-transgene lines. Our results demonstrate that plant cysteine proteases, which have the activity of increasing PM permeability, can be used to improve the plant-mediated RNAi against herbivorous insects.

**Electronic supplementary material:**

The online version of this article (doi:10.1007/s11103-013-0030-7) contains supplementary material, which is available to authorized users.

## Introduction

Plant accumulates defensive compounds (phytoalexins) to resist or evade herbivores (Gatehouse 2002). To accommodate toxins from their host plants, herbivorous insects have developed adaptive mechanisms which often involve enhanced expression of detoxification enzymes, such as P450 monooxygenases (Wittstock et al. 2004; Schuler 2011). Gossypol and related sesquiterpene aldehydes, the major phytoalexins of cotton, are distributed in root and pigmented glands of aerial organs of cotton (Tan et al. 2000). Cotton bollworm (*Helicoverpa armigera*) is a generalist herbivorous insect with cotton as its major host plant. Previously, we identified a P450 gene, *CYP6AE14*, from *H. armigera*, and its up-regulation was correlated with gossypol tolerance of bollworm larvae. When treated with piperonyl butoxide (PBO), an inhibitor of P450 monooxygenases, the bollworm larvae exhibited hypersensitivity to gossypol (Mao et al. 2007). Suppressing the expression of detoxification genes is expected to undermine bollworm defense, leading to the control of pest populations.

In eukaryotes RNA interference (RNAi) is one of the prevalent mechanisms of gene regulation. Since discovery, RNAi has seen rapid development as a powerful tool in not only fundamental research but also biotechnological applications (Cronin et al. 2009; Possemato et al. 2011). Our previous report showed that expression of the bollworm P450 gene *CYP6AE14* was repressed by RNAi through ingestion of the plants engineered to express the dsRNA (*dsCYP6AE14*), the larvae became more susceptible to gossypol (Mao et al. 2007). Further investigation demonstrated that transgenic cotton plants expressing *dsCYP6AE14* show enhanced protection from cotton bollworm feeding damage (Mao et al. 2011). The plant-triggered insect RNAi was also demonstrated with western corn rootworm (WCR); transgenic corn plant engineered to express WCR dsRNAs was more resistant to the rootworm damage (Baum et al. 2007). These findings demonstrated that plants can be armed with dsRNA to fence off insect pests.

In recent years, RNAi triggered by dsRNA ingestion has been observed in a wide range of insects. In pea aphid (*Acyrthosiphon pisum*), *C002* plays an important role in the aphid feeding on host plant; suppressing *C002* expression via plant-expressed dsRNA resulted in a significant reduction of feeding damage (Mutti et al. 2006, 2008). Oral ingestion of synthetic dsRNA caused suppression of *CYP6BG1* in diamondback moth (*Plutella xylostella*) and reduced larval resistance to the insecticide permethrin (Bautista et al. 2009). RNAi effect was observed even by spraying synthesized dsRNA solution uniformly on the newly hatched Asian corn borer larvae (Wang et al. 2011). Together, accumulating evidence indicates that RNAi is conserved in insects of different orders, including Lepidoptera, Hemiptera, Coleoptera, Diptera and Hymenoptera (Huvenne and Smagghe 2010), which makes it possible to utilize RNAi technology to control various insect pests (Gordon and Waterhouse 2007; Mao et al. 2009).

One of the problems of the plant-mediated RNAi technology is that the engineered dsRNA-expressing plants often show a mild enhancement of insect resistance, unlike the transgenic plants expressing a modified *Bacillus thuringiensis* (Bt) toxin which is highly effective against defined groups of insects (Price and Gatehouse 2008). As a new genetic tool, further investigation is needed to improve the RNAi efficiency for better crop protection. Production of effective forms of dsRNAs in plants and spreading of these silencing molecules into gut cells of insect are two key steps of plant-mediated insect RNAi. During insect ingestion, the first barrier that the food components encounter is midgut peritrophic matrix (PM), a chitin and glycoprotein layer that prevents large molecules and toxins from entering into midgut cells (Hegedus et al. 2009). It was shown that disruption of PM structure improved midgut permeability and caused adverse effects on insect (Barbehenn 2001). Since the transmission of the RNAi signal into midgut cells could be a limiting factor for the success of ingestion-triggered RNAi, it is then interesting to ask if the RNAi effect can be elevated by impairing the PM barrier.

The major digestive proteases in midgut of lepidopteran larvae are serine proteases, to which the protein components in PMs are highly resistant (Wang and Granados 2001). However, PM proteins can be hypersensitive to other types of proteases, such as plant cysteine proteases. It was reported that oral ingestion of a painpan like cysteine protease from maize (*Zea mays*), Mir1 (AF019145), caused damage to PM structures of fall armyworm, *Spodoptera frugiperda* (Pechan et al. 2002). By analysis and application of two plant cysteine proteases isolated from cotton (*G. hirsutum*) and *Arabidopsis thaliana*, respectively, we show that the cysteine proteases are able to enhance the ingestion-mediated RNAi effect of insects. Simultaneous expression of dsRNA and protease in plant provides a better protection against herbivorous insects.

## Materials and methods

### Plant and insect culture

Plants of *A. thaliana* (ecotype Col-0) were grown in pre-sterilized soil at 22 °C and at 60 % relative humidity on a 16-h-day/8-h-night cycle. Seeds were surface sterilized in 30 % (v/v) bleach containing 0.01 % Triton X-100 for 10 min, washed four times with sterile water. After synchronization at 4 °C for 72 h, the sterilized seeds were sown on the 1/2 MS agar plates, cultivated for one week and green seedlings were selected and moved to soil. Rosette leaves of ~4-week-old plants were used for wounding treatment and insect feeding tests.

Plants of cotton (*Gossypium hirsutum* cv. R15) were grown in greenhouse under 28–30 °C, 60–80 % relative humidity. Young leaves with the same conditions were used for insect feeding tests.

Cotton bollworm (*Helicoverpa armigera*) eggs were obtained from Nanjing Agricultural University and reared as previously described (Peng et al. 2005). For each feeding experiment, synchronous larvae were selected, weighed individually and divided into groups; each group contained 20–30 individuals. After feeding on different diets for indicated days, larvae were weighed and midgut was dissected and rinsed in PBS buffer twice to get rid of food particles for further analysis. Statistics of data was performed with student *t* test in the Excel Program.

### Plant transformation

The full-length open reading frames of *GhCP1* and *AtCP2* were used to replace *GUS* in pBI121 (Clontech) to generate the *Pro35S: GhCP1* and *Pro35S:AtCP2* constructs. The binary vectors harboring the desired construct were transferred into *Agrobacterium tumefaciens* strain GV3101 (for *Arabidopsis*) and LBA4404 (for *G. hirsutum*) by electroporation. Transgenic *Arabidopsis* plants were generated by a floral dip method (Clough and Bent 1998), and screened on solid plates containing 30 mg/L kanamycin. Transgenic cotton plants were generated by transformation of hypocotyl explants from *G. hirsutum* cv. R15 (Shangguan et al. 2008). After the stages of callus induction, proliferation, embryogenic callus induction, embryo differentiation, and finally plantlet regeneration, the plantlets were transferred to pots in greenhouse for further growth. Transgenic plants were screened by kanamycin selection and further confirmed by PCR for the presence of the neomycin phosphotransferase II (*NptII*) gene by specific primers (Table S1), generating a 680-bp fragment.

Genomic DNA was isolated as described (Benbouza et al. 2006). The genomic DNA (20 μg) was digested with the indicated enzyme for 16 h, separated on 0.8 % agarose gel, and transferred onto a Hybond N+ membrane (Amersham). DNA gel blot analysis of *G. hirsutum* cv R15 and transgenic cotton plants was carried out using an *NptII* fragment as probe, which was obtained by PCR using primers as described above.

### Prokaryotic expression and in vitro assay of protease activity

The full-length open reading frames of GFP, GhCP1 and AtCP2 cDNAs were amplified using *pfu* DNA polymerase and inserted into pET32a (Stratagene) between cloning sites BamHΙ and SacΙ. Each recombinant protein was fused with thioredoxin protein and His-tag, and in total, the fusion peptide is 21 kD. The recombinant GFP protein was used as control. *E. coli* BL21 (DE3) (Stratagene) was used for protein production using 0.5 mM IPTG as inducer. Bacteria were finally gathered by centrifuge at 10,000 rpm for 5 min and washed one time with double distilled water. Recombinant proteins were purified with Ni–NTA spin columns (Qiagen). Protein concentration was determined by the Bradford method with BSA as standard.

Cysteine protease activity was assayed by mixing 50 μl protein extraction (2 mg/ml of purified proteins in 50 mM Tris–HCl, pH 8.5) and 50 μl 1.5 % azocasein solution (Sigma), followed by incubation at room temperature for an indicated time. Reactions were stopped by adding an equal volume of 10 % trichloroacetic acid and incubation at room temperature for 1 h. The mixture was then centrifuged at 16,000×*g* for 10 min to remove the undigested azocasein. The supernatant was collected and mixed with an equal volume of 1 N NaOH and then the optical density was determined at 450 nm (Li et al. 2009). The supernatant of GFP (replacing the protease) reaction was used as blank.

For in vitro assay of digestion of PM proteins by GhCP1 and AtCP2, midgut PM was isolated from mid-fifth instar larvae fed on artificial diet and then thoroughly rinsed in deionized water with several changes to clean up food residue. Midgut PMs were than soaked into Tris–HCL buffer (50 mM Tris–HCl, pH 8.5) containing 2 mg/ml purified GFP, GhCP1 and AtCP2 proteins, respectively, incubating at 25 °C for 16 h. After incubation, midgut PMs were washed with deionized water several times and stained with Coomassie blue staining solution (Thermo Lot # NC 168291).

### RNA analysis

Total RNAs were isolated from *H. armigera* larvae and *Arabidopsis* plants by Trizol reagent (Invitrogen), and from cotton as described (Wu et al. 2002). For RT-PCR, the first strand cDNA was prepared using the ReverTra Ace kit (TOYOBO). Real-time RT-PCR (qRT-PCR) was performed on a Bio-Rad iCycler with iQ SYBR Green Supermix (Bio-Rad), following a two-step protocol: 95 °C for 3 min, 40 cycles of denaturation at 95 °C for 20 s and annealing/extension at 60 °C for 20 s. *Histone3* (AF024716) of cotton, *S18* (At4g09800) of *Arabidopsis* and *ACTA3b* (X97615) of bollworm were used as internal standard.

### Protein analysis

The rabbit antiserum against a CYP6AE14 fragment (150–311 amino acid residues) was raised, and the antibody was purified by binding with Protein A-Sepharose CL6B (Sigma), followed by selective elution of IgG with 50 mM glycine, pH 3.0, 0.5 mM NaCl, neutralized with 1 M Tris/HCl to pH 7.0, and used at 1:1,000 dilution.

Total proteins of the bollworm midgut were extracted and loaded onto a 10 % SDS-PAGE gel (20 μg proteins per lane). After electrophoresis, the proteins were electrotransferred to a Hybond-C membrane (Amersham). Blots were incubated with the primary antibody for 3–4 h, than incubated with Horseradish Peroxidase (HRP)-conjugated anti-rabbit antiserum as the secondary antibody for 45 min. The blot was developed using enhanced chemiluminescence (ECL) detection solution (Tiangen) and exposed to X-ray films.

### Analysis of sesquiterpene aldehydes

Total sesquiterpene aldehydes were quantitated with a phloroglucinol/HCl assay (Liu et al. 1999). To detect gossypol in bollworm midgut, the midgut was washed 3 times in physiological saline, and homogenates of ten individual midguts were extracted with 70 % acetone for 30 min. After centrifugation, an equal volume of the reagent (1 % phloroglucinol, 2 N HCl in 95 % ethanol) was added to the acetone extract, and incubated at 55 °C for 5 min. The absorbency at 555 nm was immediately measured. Standard curve was prepared with gossypol (Sigma). For cotton leaf samples, 500 mg cotton leaves (fresh weight) were immersed in liquid nitrogen, ground into a fine powder, followed by extraction and detection as described above.

### Virus infection

*Dendrolimus punctatus* cytoplasmic polyhedrosis virus (DpCPV) was obtained from Wuhan Institute of Virology, Chinese Academy of Sciences. The viruses were diluted into 10^7^ polyhedron-shaped inclusion bodies (PIB)/mL in PBS buffer, and 2 μl of the diluted suspension were dropped onto a 2-mm^2^ area of a cotton leaf. After being fed with the DpCPV stained leaves for 10 h, larvae were transferred to artificial diet or unstained fresh leaves. After 2 days of feeding, larval midgut was taken and analyzed by qRT-PCR to detect DpCPV genes *S1* (AY163247), *S3* (AY167578) and *S4* (AF542082).

## Results

### Expression pattern of *GhCP1* and *AtCP2* in plants

**Fig. 1:**
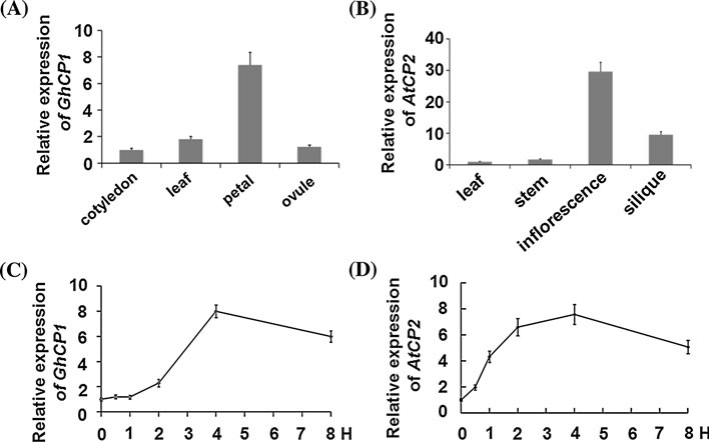
Expression patterns of *GhCP1* and *AtCP2*. **a** *GhCP1* expression in cotton (*G. hirsutum*). Cotyledon of 1-week-old seedlings, and leaf (the second leaf from *top*), petal and ovule (day 3 post-anthesis) of adult plant (12 weeks old) were analyzed; **b** *AtCP2* expression in leaf, stem, inflorescence and silique of 4-week-old plants of *A. thaliana*; Expression of *GhCP1* (**c**) and *AtCP2* (**d**) after wounding. The 4–week-old cotton plant and the *Arabidopsis* leaves were treated by wounding for indicated times. The expressions of *GhCP1* in cotyledon and *AtCP2* in leaf were set to 1. In wounding treatment, the expression level just before treatments was set to 1. Each investigation had at least three biological repeats, *error ba*rs represent standard deviation (SD)

To isolate cysteine proteases from cotton (*G. hirsutum*), we searched database for genes encoding proteins with sequence similarities to MirCP1 (AF019145), a cysteine protease from maize (Pechan et al. 2002). Three of them, *GhCP1* (CAE54307), *GhCP2* (AY171099) and *GhCP3* (CAE54306) were identified, and they show 48, 37 and 28 % amino acid sequence identities, respectively, to MirCP1. Analysis with RT-PCR and quantitative RT-PCR (qRT-PCR) showed that *GhCP1* had a high level of expression in petal only, and *GhCP3* was also highly expressed in petal, with a low level of transcripts present in leaf, cotyledon and ovule, whereas *GhCP2* was expressed widely in cotton plants (Fig. 1a; Fig. S1). In *A. thaliana* genome, *AtCP1* (At4g11310) and *AtCP2* (At4g11320) encode putative cysteine proteases and share high sequence amino acid sequence identities (45 and 44 %, respectively) to MirCP1. Although located side by side in genomic locus, *AtCP1* and *AtCP2* exhibited different expression patterns. *AtCP1* was expressed exclusively in inflorescence (Fig. S2), whereas *AtCP2* was highly expressed in seedling, root and inflorescence, with a low level of expression also detected in stem (Fig. 1b; Fig. S2).

*GhCP1* and *AtCP2* were then selected for further analyses. Because plant defense reactions against herbivores often also respond to wounding (Koo and Howe 2009), we examined the change of expression levels of *GhCP1* and *AtCP2* in response to mechanical damage. When leaves of the two-week-old cotton seedlings were wounded, the abundance of *GhCP1* transcripts was increased and peaked at 4 h after the treatment (Fig. 1c). In *Arabidopsis*, *AtCP2* responded to wounding treatment quickly; an evident induction of *AtCP2* expression was observed at 2 h post-treatment and the high level of transcripts was maintained for at least 8 h (Fig. 1d).

### Proteolytic activities of GhCP1 and AtCP2

**Fig. 2:**
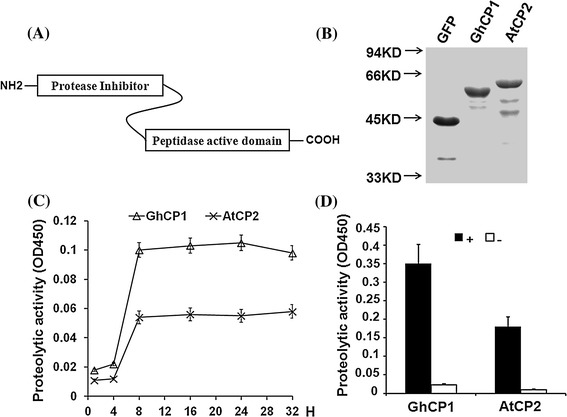
Proteolytic activity of GhCP1 and AtCP2. **a** Sketch of GhCP1 and AtCP2 domains; **b** SDS-PAGE of purified proteins. His-tag fusion proteins of GFP, GhCP1 and AtCP2 were expressed in *E. coli*; **c** Proteolytic activity of purified His-GhCP1 and His-AtCP2. Purified proteins were added in reaction buffer and incubated at room temperature for indicated times as described in “Materials and methods”; **d** Effect of gut fluids on proteolytic activity of GhCP1 and AtCP2. Purified His-GhCP1 and His-AtCP2 proteins (final concentration: 2 mg/ml) were added to reaction buffer and incubated at room temperature for 1 h. +1 μl of gut fluid of 5th instar larvae was added to 100 μl reaction buffer; −no gut fluid added. Data are shown as mean ± SD

Cysteine protease harbors two domains: the N-terminal protease inhibitor domain and the C-terminal peptidase activation domain (Fig. 2a) (McGrath 1999). It has been reported that activation of cysteine proteases requires the removal of N-terminal inhibitor domain (Bryan 2002). To analyze the proteolytic activities of GhCP1 and AtCP2, we expressed His-tag fusion proteins of both in *Escherichia coli*, respectively, and the fused green fluorescent protein (GFP) (His-GFP) was used as a negative control (Fig. 2b). When using azocasein as substrate, the proteolytic activities of GhCP1 and AtCP2 were extremely weak under standard conditions. However, the proteolytic activities of His-GhCP1 and His-AtCP2 were remarkably increased when midgut fluids from bollworm were added into the reaction mixture (Fig. 2c, d). These results suggest that GhCP1 and AtCP2 can be activated by insect midgut fluids, implying a role of plant cysteine proteases in plant–insect interactions.

### Elevation of bollworm PM permeability by cysteine protease treatment

**Fig. 3:**
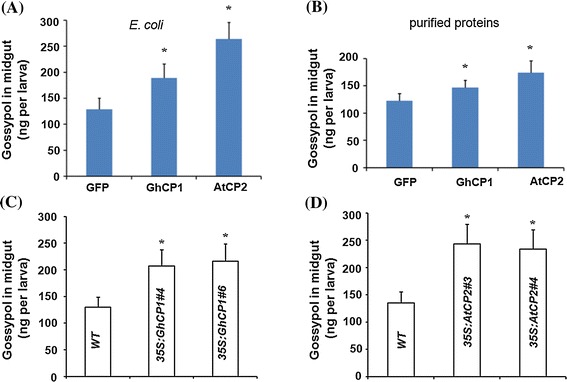
The effects of cysteine protease on bollworm midgut PM permeability to gossypol. Varied gossypol accumulation in midgut after bollworm ingestion of cysteine proteases. The 3rd instar larvae were fed with artificial diet supplemented with *E. coli* expressing His-tag fusion proteins of GFP, GhCP1 or AtCP2, respectively (**a**), or with the purified fusion proteins (**b**) for 8 h, and then transferred to gossypol (1 mg/g)-supplemented diet for another 16 h; gossypol content in midgut was measured by a phloroglucinol/HCl assay. To 10 g artificial diet, precipitates of 50 ml liquid culture of *E. coli* cells (OD 1.0), or purified His-GhCP1, His-AtCP2 or His-GFP proteins (10 mg each), respectively, were added; Accumulation of gossypol in midgut cells of the bollworms that were pre-fed with *35S:GhCP1* (**c**) or *35S:AtCP2* (**d**) plant leaves. The 3rd instar larvae were fed on wild-type (WT) or transgenic *Arabidopsis* leaves for 2 days, then transferred to diet containing gossypol (1 mg/g) for another day. Data are shown as mean ± SD. * *p* < 0.05

To examine the effect of cysteine proteases on midgut PM structure of cotton bollworm, the PM was isolated from 5th instar larvae and incubated with 2 mg/ml purified GFP, GhCP1 and AtCP2, respectively. After incubation, PM proteins were visualized by Coomassie blue staining. We found that the staining became much paler if the PMs were treated with GhCP1 or AtCP2 (Fig. S3), indicating that GhCP1 and AtCP2 could affect the PM structure by digesting the PM proteins. Next, we investigated whether ingested GhCP1 and AtCP2 would affect PM permeability. The 3rd instar larvae were fed with artificial diet supplemented with *E. coli* cells expressing His-GhCP1, His-AtCP2, or His-GFP, respectively. After 8 h, the larvae were transferred to artificial diet containing 1 mg/g gossypol. The amount of gossypol in midgut was measured after 16 h. We found that the larvae pre-fed with His-GFP *E. coli* accumulated 129 ng gossypol per larva in their midgut, whereas those with His-GhCP1 or His-AtCP2 *E. coli* accumulated 182 and 241 ng per larva, respectively, significantly higher than the control (Fig. 3a). Similar feeding assay results were obtained when the bacterial cells were replaced by the purified fusion proteins (Fig. 3b).

We then generated transgenic *Arabidopsis* plants expressing *GhCP1* or *AtCP2*, under the constitutive CaMV *35S* promoter. RT-PCR analyses were performed to select the transgenic lines with high expression levels of *GhCP1* or *AtCP2* (Fig. S4). Leaves of two *35S:GhCP1* lines (#4 and #6) and two *35S:AtCP2* lines (#3 and #4) were used for subsequent feeding assay. It has been reported that cysteine proteases may, in some cases, have insecticidal effects (Pechan et al. 2002; Mohan et al. 2008). To see whether plant over-expressing *GhCP1* and *AtCP2* would have an adverse effect on bollworm growth, we fed the 3rd instar larvae with wild type, *35S:GhCP1* or *35S:AtCP2* plant leaves. No obvious difference of larvae growth was seen and the weight increase of the tested larvae groups was similar after a four-day feeding (Fig. S5). However, when the *35S:GhCP1* or the *35S:AtCP2* pre-fed bollworms were transferred to gossypol-containing diet, higher contents of gossypol were detected in midgut compared to the control (Fig. 3c, d). These data demonstrate that the cysteine protease, after ingestion by bollworms, increased PM permeability to gossypol.

**Fig. 4:**
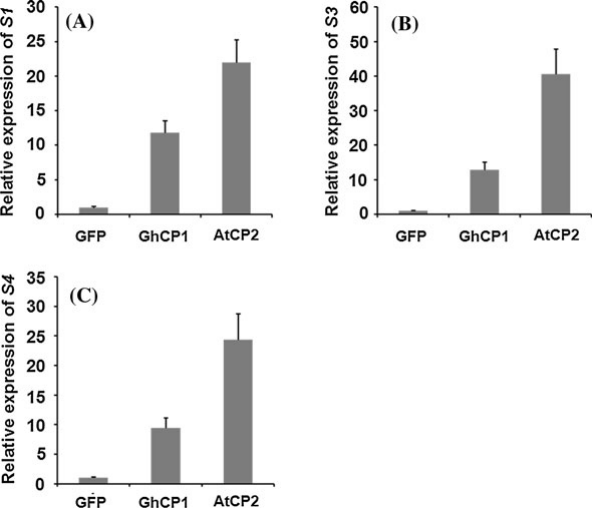
Expression of DpCPV genes in bollworm midgut. The 3rd instar larvae were fed with artificial diet supplemented with *E. coli* cells expressing indicated fusion proteins for 2 days, and then infected with DpCPV. After 2 days, total RNAs from midgut were extracted and subject to qRT-PCR to detect the expression of DpCPV genes *S1* (AY163247) (**a**), *S3* (AY167578) (**b**) and *S4* (AF542082) (**c**). Expression level of DpCPV genes in the larvae of the GFP group was set to 1. qRT-PCR analysis was biologically repeated for at least three times, *error bars* represent SD

Elevation of PM permeability was reported to result in augmented virus infection (Hegedus et al. 2009). *Dendrolimus punctatus* cytoplasmic polyhedrosis virus (DpCPV) is an important pathogen of *Dendrolimus punctatus* walkers (Lepidoptera), and it also infects midgut epithelial cells of other lepidopteran species, including cotton bollworm (Belloncik 1989; Zhao et al. 2004). We then tested whether the sensitivity of bollworm midgut to viral infection could be changed by cysteine proteases. The 3rd instar larvae were fed with artificial diet mixed with *E. coli* cells expressing the fusion cysteine protease for one day and then infected with DpCPV. Two days later, quantitative real-time PCR (qRT-PCR) was performed to detect the abundance of DpCPV genes *S1* (AY163247), *S3* (AY167578) and *S4* (AF542082), which encode major core proteins, in bollworm midgut. We found that the transcript levels of *S1* in the larvae fed with His-GhCP1 or His-AtCP2 were 12- and 22-fold higher, respectively, than those fed with His-GFP. Similar elevation of the *S3* and *S4* transcript levels were also observed in the larvae previously ingested the GhCP1- or AtCP2-supplemented diet (Fig. 4). These data demonstrate that the bollworm larvae became more susceptible to the virus infection after up-taking the plant cysteine protease.

### Ingestion of cysteine protease enhances plant-mediated RNAi

**Fig. 5:**
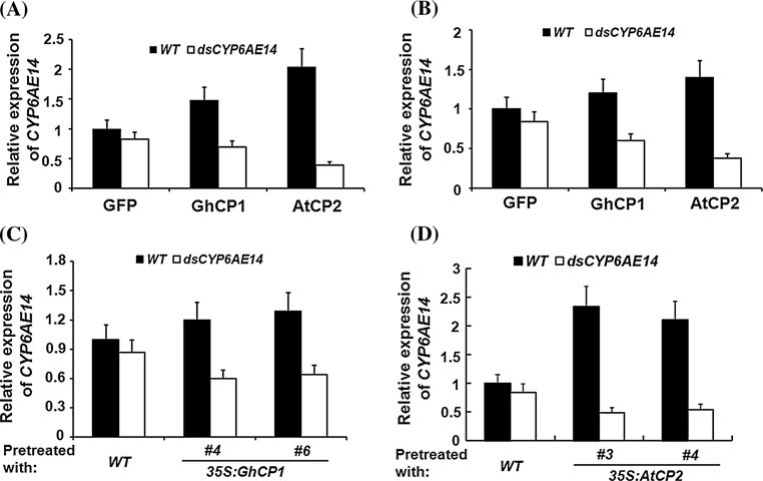
The effect of cysteine protease on plant-mediated bollworm RNAi. The 3rd instar larvae pre-fed with different diets for 2 days were transferred to wild-type (WT) or *35S:dsCYP6AE14* *Arabidopsis* leaves for 16 h, *CYP6AE14* transcript level in midgut was analyzed by qRT-PCR. Larvae were pre-fed with artificial diet supplemented with *E. coli* cells expressing His-tag fusion proteins of GFP, GhCP1 and AtCP2, respectively (**a**), or with purified fusion proteins (**b**), for 2 days; Larvae were pre-fed with *WT*, *35S:GhCP1*(**c**) or *35S:AtCP2* (**d**) *Arabidopsis* leaves, respectively, for 2 days. qRT-PCR analysis was biologically repeated for at least three times, *error bars* represent SD

DpCPV, which is a dsRNA virus, contains 10 separate fragments of dsRNA (Zhao et al. 2004). Elevated DpCPV infection after cysteine protease ingestion led us to propose that the protease might also promote traverse of dsRNA molecules across the midgut PM, which would result in enhanced efficiency of RNAi induced by ingested dsRNA. To test this possibility, 3rd instar larvae were fed with artificial diet supplemented with the His-GFP, His-GhCP1 and His-AtCP2 *E. coli* cells, for 2 days. The larvae were then transferred to leaves of transgenic *Arabidopsis* plants expressing the dsRNA against the bollworm P450 gene *CYP6AE14* (Mao et al. 2007). While the transcript level of *CYP6AE14* was moderately decreased in the control (His-GFP) group, a strong repression of *CYP6AE14* expression occurred in the larvae pre-treated with His-GhCP1 or His-AtCP2 (Fig. 5a). Similar results were obtained when the purified fusion proteins of His-GhCP1 and His-AtCP2 (Fig. 5b), or the transgenic *35S:GhCP1* and *35S:AtCP2* *Arabidopsis* leaves were used for the pre-treatment (Fig. 5c, d).

### Cotton plants co-expressing *dsRNA* and cysteine protease exhibit enhanced insect-resistance

**Fig. 6:**
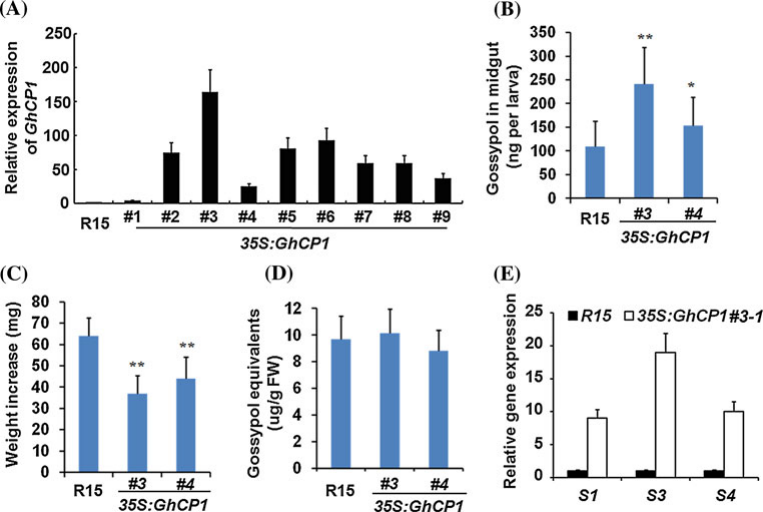
Effects of cotton overexpressing *GhCP1* on bollworm larvae. **a** Expression of *GhCP1* in cotton. Nine *35S:GhCP1* lines were analyzed by qRT-PCR, *GhCP1* expression in wild-type cotton (R15) leaves was set to 1; Accumulation of gossypol in midgut and weight increase of tested larvae. 3rd instar larvae were fed on leaves of wild-type (R15) and *35S:GhCP1* (two independent lines: #3 and #4) cotton for 7 days. Gossypol accumulation in midgut (**b**) and bollworm weight increase (**c**) were recorded. * *P* < 0.05, ** *P* < 0.01; **d** The level of gossypol equivalents in tested cotton plant leaves; **e** Expression of DpCPV genes *S1* (*AY163247*), *S3* (*AY167578*) and *S4* (*AF542082*) in bollworm midgut. 3rd instar larvae were fed with wild-type (R15) or *35S:GhCP1#3*-*1* cotton leaves for one day and then infected with DpCPV; after 2 days, total RNAs from midguts were extracted and subject to qRT-PCR. The expression of DpCPV genes in the larvae pre-fed with wild-type leaves was set to 1. qRT-PCR analysis was biologically repeated for at least three times, *error bars* represent SD

The *35S:GhCP1* construct was then introduced into cotton (*G. hirsutum*) plants. Nine transgenic lines were obtained based on PCR genotyping and southern blot (Fig. S6). Two lines (#3 and #4) were selected for insect feeding. Gene expression analyses revealed that the transcript level of *GhCP1* was substantially elevated in *35S:GhCP1* plants in comparison with the wild-type (Fig. 6a). After one week of feeding, the average content of gossypol in midgut from the larvae fed with *35S:GhCP1* lines (*#3* and *#4*) was about 242 and 151 ng per larva, respectively, whereas the larva fed with wild-type cotton leaves accumulated only 108 ng gossypol in midgut (Fig. 6b). Consistently, larval growth of the *35S:GhCP1* group was retarded compared to the control (Fig. 6c). The difference in weight gain was unlikely due to gossypol contents in plants since the two *35S:GhCP1* lines accumulated nearly the same level of gossypol equivalents as the wild-type (Fig. 6d). T1 generation of *35S:GhCP1#3*, germinated from seeds of the T0 plant, was analyzed by RT-PCR, and *35S:GhCP1#3*-*1* with a high expression level of *GhCP1* (Fig. S7a) was selected for insect feeding test. When 3rd instar larvae fed with *35S:GhCP1#3*-*1* leaves for 5 days, higher gossypol contents were detected in midgut and the larvae exhibit stunted growth compared to the control (Fig. S8). We then tested larval susceptibility to DpCPV. After 3rd instar larvae were fed with the cotton leaves for one day, infection by DpCPV was performed. Two days later, *S1*, *S3* and *S4* transcript levels were about 8, 10 and 17 fold higher in the larvae fed on the *35S:GhCP1* than those on the wild-type cotton leaves, according to qRT-PCR analysis of midgut (Fig. 6e).

**Fig. 7:**
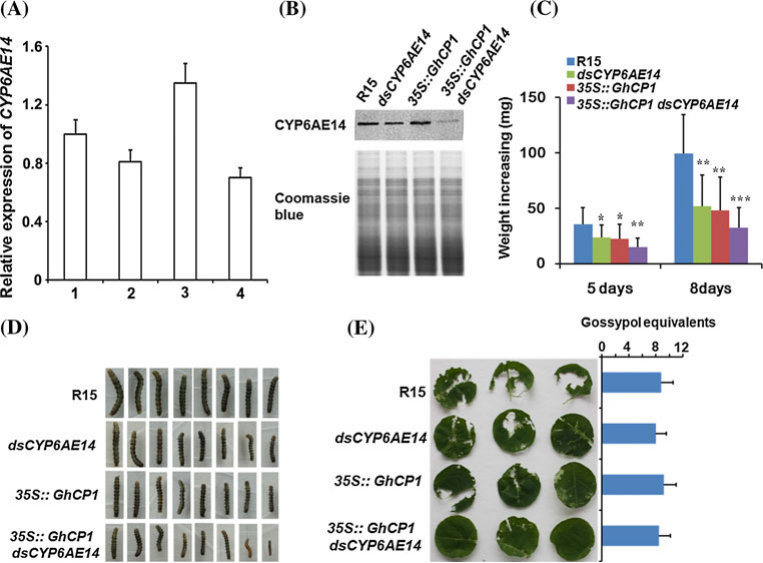
Enhanced bollworm resistance of cotton co-overexpressing *GhCP1* and *dsCYP6AE14*. **a** Expression level of *CYP6AE14*. 3rd instar larvae were fed with the indicated leaves (1: wild-type (R15), 2: *dsCYP6AE14*, 3: *35S:GhCP1* and 4: *35S:GhCP1 dsCYP6AE14*) for 16 h. *CYP6AE14* expression in midgut of the larvae fed with wild-type (R15) leaves were set to 1; **b** Accumulation of CYP6AE14 protein in midgut. 3rd instar larvae were fed with wild-type (R15), *dsCYP6AE14*, *35S:GhCP1* and *35S:GhCP1 dsCYP6AE14* cotton leaves for 2 days. Midgut proteins were detected with an antiserum against CYP6AE14; **c** Weight increase of the 2nd instar larvae fed with the indicated cotton leaves for 5 and 8 days. * *p* < 0.05; ** *p* < 0.01; *** *p* < 0.001; **d** The 2nd instar larvae fed with the indicated cotton leaves for 5 days; **e** The 2nd instar larvae previously fed with the indicated cotton leaves for 5 days were transferred to fresh leaves for another day, then the leaf damage after 1 day of feeding by bollworms was shown (*left*); gossypol equivalents in cotton leaves were displayed in the right. *Error bars* represent SD

Recently we reported that transgenic cotton plants expressing dsRNAs against *CYP6AE14* (*dsCYP6AE14*) exhibited higher resistance to bollworms (Mao et al. 2011). If we could increase gossypol content in midgut by attenuating the PM, and at the same time block the bollworm gossypol detoxification pathway by RNAi, then gossypol would be more potent against bollworm. To achieve this, we crossed the *35S:GhCP1* (line *#*3) to the *dsCYP6AE14* (line #6-3) (Mao et al. 2011) to generate cotton plants co-expressing *GhCP1* and *dsCYP6AE14*. F1 plants were examined by RT-PCR, and seven individuals with high expression levels of both *GhCP1* and *dsCYP6AE14* were selected (Fig. S7b). In larvae fed on *dsCYP6AE14* cotton leaves for 16 h the *CYP6AE14* expression level in midgut was decreased, and the suppression was much severer in the larvae fed on the *35S:GhCP1* *dsCYP6AE14* co-expression cotton leaves (Fig. 7a). After 2 days of feeding, accumulation of CYP6AE14 protein was reduced in larvae fed on *dsCYP6AE14* cotton leaves and again the reduction was more obvious if the larvae were placed on the *35S:GhCP1* *dsCYP6AE14* leaves (Fig. 7b). After 5 or 8 days of feeding, respectively, retarded growth was observed in the larvae fed on all three types (*35S:GhCP1*, *dsCYP6AE14* and *35S:GhCP1* *dsCYP6AE14*) of transgenic cotton plant leaves (Fig. 7c, d), but the larvae on the *35S:GhCP1* *dsCYP6AE14* double-transgene leaves were smaller than those on either the *35S:GhCP1* or the *dsCYP6AE14* single-transgene leaves.

To examine whether retarded growth was accompanied by reduced plant consumption, the 2nd instar larvae were placed on the transgenic or the wild-type cotton leaves for 5 days, and then transferred to fresh leaves of the respective plant. The damage of leaves after one day of feeding was scored. We found that the *35S:GhCP1* *dsCYP6AE14* cotton leaves were the least damaged, and the gossypol equivalents among the tested cotton leaves were similar (Fig. 7e). These results demonstrate that combination of *dsCYP6AE14* with *35S:GhCP1* provides a better protection of cotton plants from bollworm damage than either transgenes alone.

## Discussion

Plant genome harbors a large gene family encoding cysteine proteases. In *A. thaliana* genome, for example, there are at least 32 papain-like cysteine proteases (Simpson 2001), which are believed to play an important role in plant-pest interactions (Shindo and Van der Hoorn 2008). Mir1, a papain-like cysteine protease from maize, was reported to have an adverse effect on fall armyworm caterpillars by destroying its PM structures (Pechan et al. 2002); retarded larval growth was observed when different lepidopteran caterpillars were fed on papain-containing leaves (Konno et al. 2004). In this investigation, we analyzed two cysteine proteases, *GhCP1* from cotton and *AtCP2* from *Arabidopsis*, and tested their effect on cotton bollworm midgut PM permeability to gossypol and the ingestion-triggered RNAi. In vitro assay revealed that GhCP1 and AtCP2 did affect PM structure by digesting PM proteins though the effect is mild. Although cysteine proteases are reported to have insecticidal activities (Pechan et al. 2002; Mohan et al. 2008), in this investigation overexpressing either *GhCP1* or *AtCP2* in *Arabidopsis* did not exhibit an obvious effect on bollworm growth. However, the growth inhibition was observed with the larvae fed on the *35S:GhCP1* cotton. One explanation to this difference is that cotton plants accumulate high levels of gossypol and related sesquiterpene aldehydes which function as phytoalexins; the increased level of cysteine proteinase in *35S:GhCP1* cotton leaves attenuated the PM barrier, resulting in enhanced traverse of gossypol across the PM and the elevated accumulation of the phytotoxin in midgut cells. Furthermore, in addition to its activity toward PM, the plant cysteine protease may also disturb insect detoxification enzymes, resulting in slowed degradation of gossypol, and this requires further investigation.

Bt-crops, expressing genes coding for insecticidal crystalline proteins from *Bacillus thuringiensis* (*Bt*), have achieved a great success in both economical and ecological aspects (Qaim and Zilberman 2003; Wu et al. 2008). However, the reported insect resistance to *Bt* toxins and outbreaks of non-target pests, particularly the sucking pests, have become a concern of continuous and wide planting of *Bt* crops (Bravo and Soberon 2008; Tabashnik et al. 2008; Lu et al. 2010). RNAi technology offers a new and more selective strategy for plant protection, as it can be designed to specifically down-regulate gene(s) of the insect that damages the host plant. As a recently emerged strategy, dsRNA transgenic plant seemed less effective at present time when compared to Bt-crops, thus optimizations are needed before wide application. In food-mediated insect RNAi, transmission of dsRNAs from plant to insect gut cells is an important step that determines the efficiency of RNAi, and PM in midgut is the major barrier to prevent the silencing signaling molecules from entering midgut epithelial cells. To overcome this barrier, we co-expressed cysteine protease with dsRNA in plant. The PM sensitive plant cysteine proteases used in this study may exert two effects on the insects: crippling the PM barrier and enhancing the RNAi effect. Thus our work takes an important step in optimizing RNAi-based technology for plant protection. Furthermore, the cysteine proteases may also play a role in affecting various proteins in midgut, disturbing the insect digestion and even defense systems, this is worthy of detailed investigation.

Plant secondary metabolites play an important ecological role in ecosystem, particularly in mediating plant–insect interactions. Many plant secondary metabolites are taxon-specific, and are also termed specialized metabolites (Howe and Jander 2008). To successfully live on host plants, herbivorous insects have developed distinct pathways to accommodate these phytoalexins (Schuler 2011). As we reported recently, cotton bollworm responds to gossypol by elevating expression levels of P450 monooxygenase genes, including *CYP6AE14*, and such dynamic levels of detoxification enzymes are important for the insect adaptation (Tao et al. 2012). In this investigation, we found that when bollworms were fed on the *35S:GhCP1* *dsCYP6AE14* co-expressing cotton plants, the high level of GhCP1 led to increased gossypol accumulation in bollworm midgut and severer suppression of *CYP6AE14* expression. The co-expressing cotton plants showed a better protection from bollworm feeding than either of the single transgene and the wild-type cotton lines. Thus the “double-edged sword” armed with cysteine protease and dsRNA renders the plant secondary metabolites more potent in protecting plant against invading pests.

## Electronic supplementary material

Below is the link to the electronic supplementary material.

Supplementary material 1 (DOCX 984 kb)
