# Supplementary material for: Cysteine protease enhances plant-mediated bollworm RNA interference
Source: Plant Mol Biol. 2013 Mar 4;83(1):119–29. doi: 10.1007/s11103-013-0030-7 (PMC3755213; doi:10.1007/s11103-013-0030-7)
Supplement: Supplementary file 1 — Supplementary material 1 (DOCX 984 kb) [file 11103_2013_30_MOESM1_ESM.docx]

Supplementary data for the article submitted to Plant Molecular Biology

“Cysteine protease enhances plant-mediated bollworm RNA interference”

by

Ying-Bo Mao, Xue-Yi Xue, Xiao-Yuan Tao, Chang-Qing Yang, Ling-Jian Wang, Xiao-Ya Chen*

*Corresponding author: Xiao-Ya Chen. National Key Laboratory of Plant Molecular Genetics, Institute of Plant Physiology and Ecology, Shanghai Institutes for Biological Sciences, Chinese Academy of Sciences, 300 Fenglin Road, Shanghai 200032, P. R. China. email: xychen@sibs.ac.cn

**Table S1.** Primers used in this investigation.

| Gene (Accession no.) | Primer | Purpose |
| --- | --- | --- |
| GhCP1 (CAE54307) | 5-GGG GGATCC ATGGAATTAACCCTTCTTTTC-3' | amplify full-length open reading frames of GhCP1 |
|  | 5'-GGG GAGCTC GCAATGAATTCAAGCACTGC-3' |  |
|  | 5'-ATCAGGAGTTTTTAATGGTG-3' | RT-PCR and qRT-PCR |
|  | 5'-GCAATGAATTCAAGCACTGC-3' |  |
| GhCP2 (AY171099) | 5'-CACTGATTTACCATATGGTG-3' | RT-PCR |
|  | 5'-AGGCAATAATCATAGAATTC-3' |  |
| GhCP3 (CAE54306) | 5'-GGATTATCAAGAATTCATGG-3' | RT-PCR |
|  | 5'-CTGTTCGTAAAAGAAGAGCTC-3' |  |
| AtCP1 (At4g11310) | 5'-AGCCTGTGACTGCCGTTAT-3' | RT-PCR |
|  | 5'-TTGCCCTCCATACAAATCC-3' |  |
| AtCP2 (At4g11320) | 5'-GGG GGATCC ATGGGTTATGCTAAATCAGC-3' | amplify full-length open reading frames of AtCP2 |
|  | 5'-GGG GAGCTC TAGGCAACCGAAACTTTATCC-3' |  |
|  | 5'-AATCGGGAGTGTTTGACG-3' | RT-PCR and qRT-PCR |
|  | 5'-ATGTTGCGAGCCATCTTC-3' |  |
| S18 (At4g09800) | 5’-CCAGCGATCGTTTATTGCTT-3’ | RT-PCR and qRT-PCR |
|  | 5’-AGTCTTTCCTCTGCGACCAG-3’ |  |
| GFP (AAF65344) | 5'-Atggtagatctgactagtaaag-3' | amplify full-length open reading frames of GFP |
|  | 5'-TTTGTATAGTTCATCCATGC-3' |  |
| NPTII (AFM76957) | 5’-GGCGATACCGTAAAGCACGAGGAA-3’ | RT-PCR |
|  | 5’-GCTATGACTGGGCACAACAGACAAT-3’ |  |
| Histon3 (AF024716) | 5’-GGCATACCTTGTGGGTCTTTTTGA-3’ | qRT-PCR |
|  | 5’-CTACCACTACCATCATGGC-3’ |  |
| Actin (X97615.1) | 5'-AAGTTGCTGCGCTGGTAGTAG-3' | qRT-PCR |
|  | 5'-AGTTCGTAGGACTTCTCCAGG-3' |  |
| CYP6AE14 (DQ986461) | 5'-GATTCAGAACTCTTCCACCAG-3' | qRT-PCR |
|  | 5'-TGGCATCTCCGAAAGGCACTC-3' |  |
| DpCPV-S1 (AY163247) | 5'-TGGTAAGGGAGTTGATGCC-3' | qRT-PCR |
|  | 5'-GAGCCGCGACGTTAGAGTA-3' |  |
| DpCPV-S3 (AY167578) | 5'-GATAGGACCCATAACCAAC-3' | qRT-PCR |
|  | 5'- ATCTACTTTCCAAGCCATAC-3' |  |
| DpCPV-S4 (AF542082) | 5'-ACAAATAACTGCCAACTCG-3' | qRT-PCR |
|  | 5'-ACCAACGAAAGTGATGTCC-3' |  |


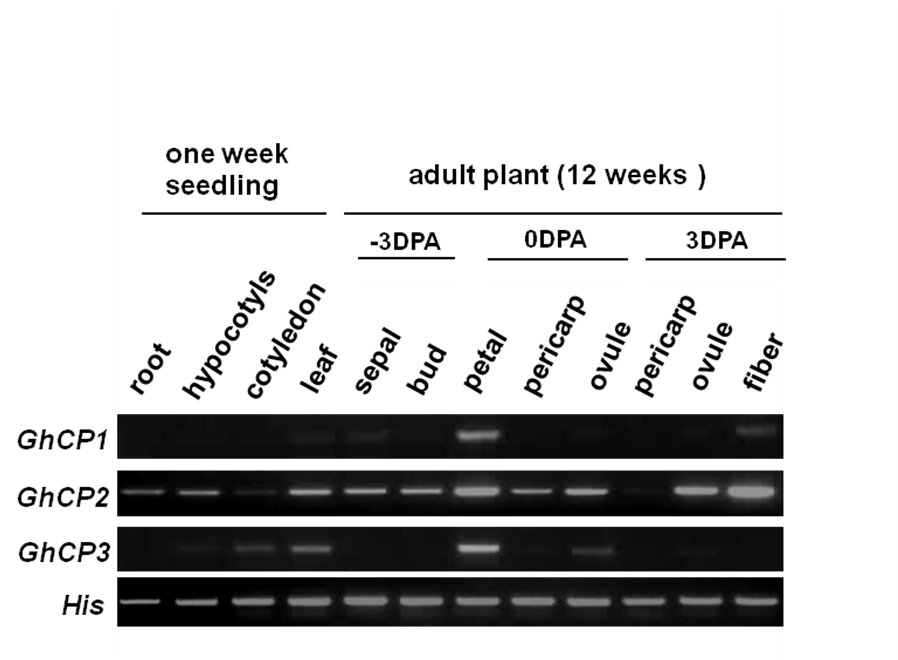


**Figure S1.** Expression of *GhCP1*, *GhCP2* and *GhCP3* in cotton plant.

RT-PCR analysis of *GhCP1*, *GhCP2* and *GhCP3* expressions in root, hypocotyl and cotyledon of one-week-old seedlings and in indicated tissues of adult plant (12 weeks old) of cotton (*G. hirsutum*). DPA: days post-anthesis. Expression of *Histon3* (*His*) was analyzed as a reference.


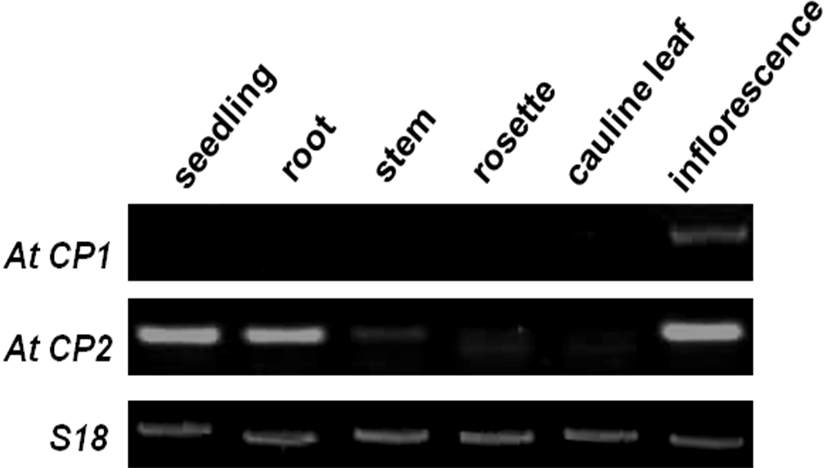


**Figure S2.** Expression of *AtCP1* and *AtCP2* in *Arabidopsis* plant.

RNAs were extracted from different tissues and subjected to RT-PCR analyses. Expression of *S18* was used as a reference.


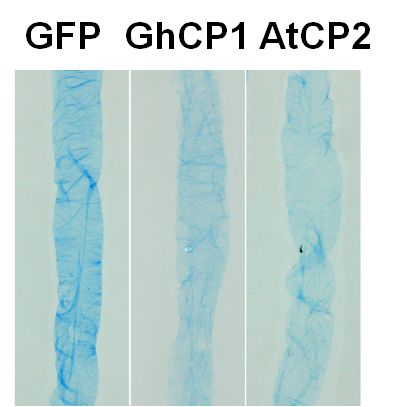


**Figure S3.** *In vitro* treatment of bollworm midgut peritrophic matrix (PM) by recombinant GhCP1 and AtCP2. Midgut PMs were incubated with 2 mg/ml purified GFP, GhCP1 and AtCP2 recombinant proteins respectively, in Tris-HCL buffer (50 mM Tris–HCl, pH 8.5) at 25 °C for 16 hours. Proteins in midgut PMs were then visualized by Coomassie blue staining. Note that after treatments by GhCP1 or AtCP2, the PM staining became much paler, indicating that the PM proteins were digested.


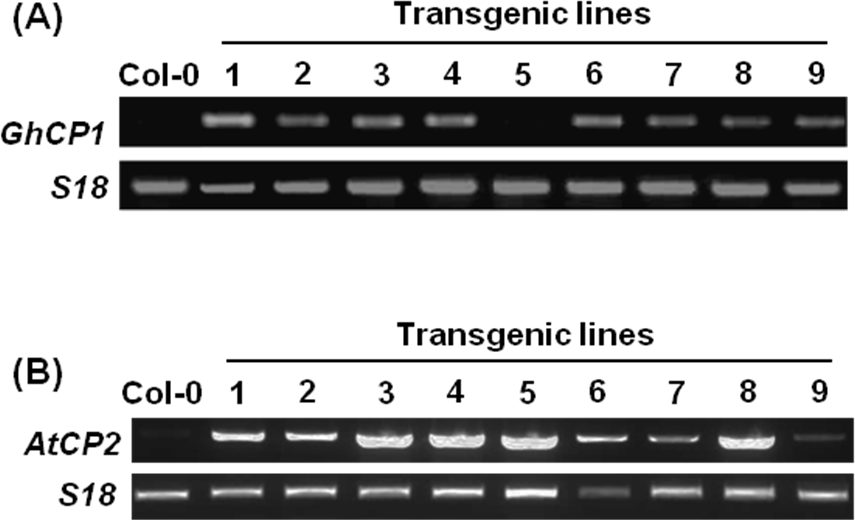


**Figure S4.** Expression of *GhCP1* and *AtCP2* in transgenic 35S:*GhCP1* (**a**) and *35S:AtCP2* (**b**) *Arabidopsis* plants.

Total RNAs form leaves were extracted and subjected to RT-PCR analyses. Expression of *S18* was used as a reference.


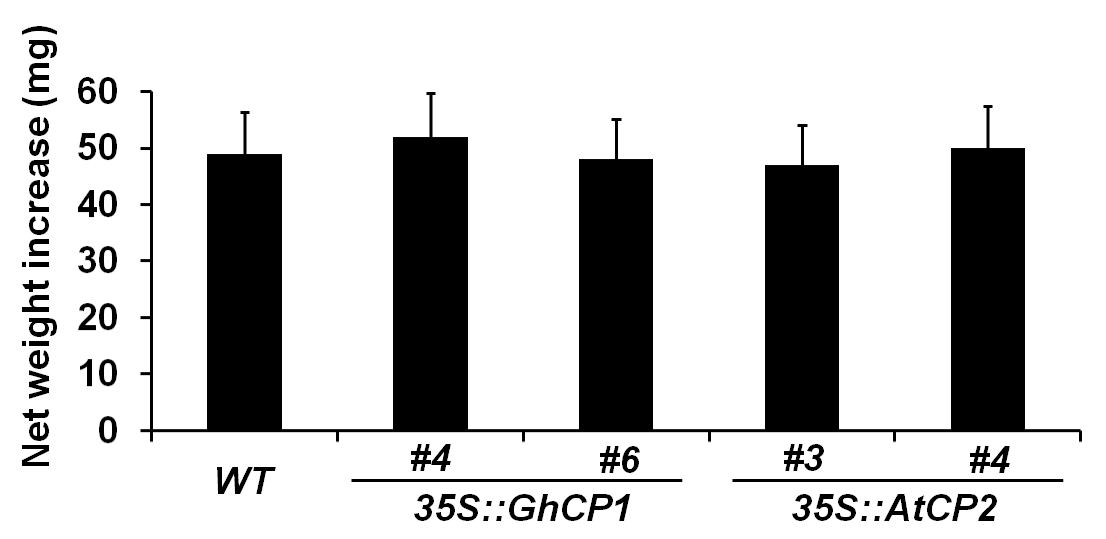
 **Figure S5.** Net weight increase of the larvae fed on indicated plants. The 3rd instar larvae were fed with leaves of wild-type (WT) or transgenic *Arabidopsis* plants (4 weeks old) for 4 days. Error bars represent standard deviation.


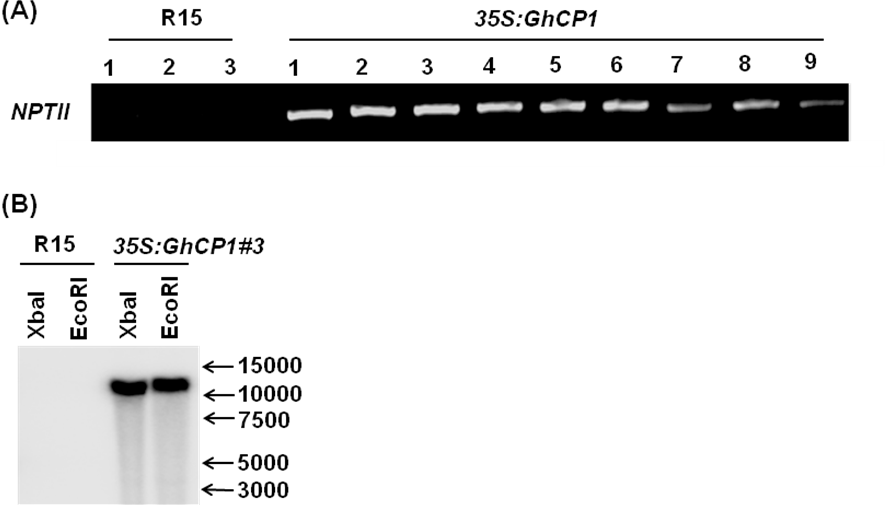


**Figure S6.** Identification of transgenic cotton lines.

**a** PCR analysis of *NPTII* in wild-type (R15) and *35S:GhCP1* transgenic cotton lines. Genomic DNA was isolated from leaves*.* **b** Southern blot analyses of transgenic cotton plants. Genomic DNA of wild-type and *35S:GhCP1* (*#3*) leaves was digested by enzymes as indicated.


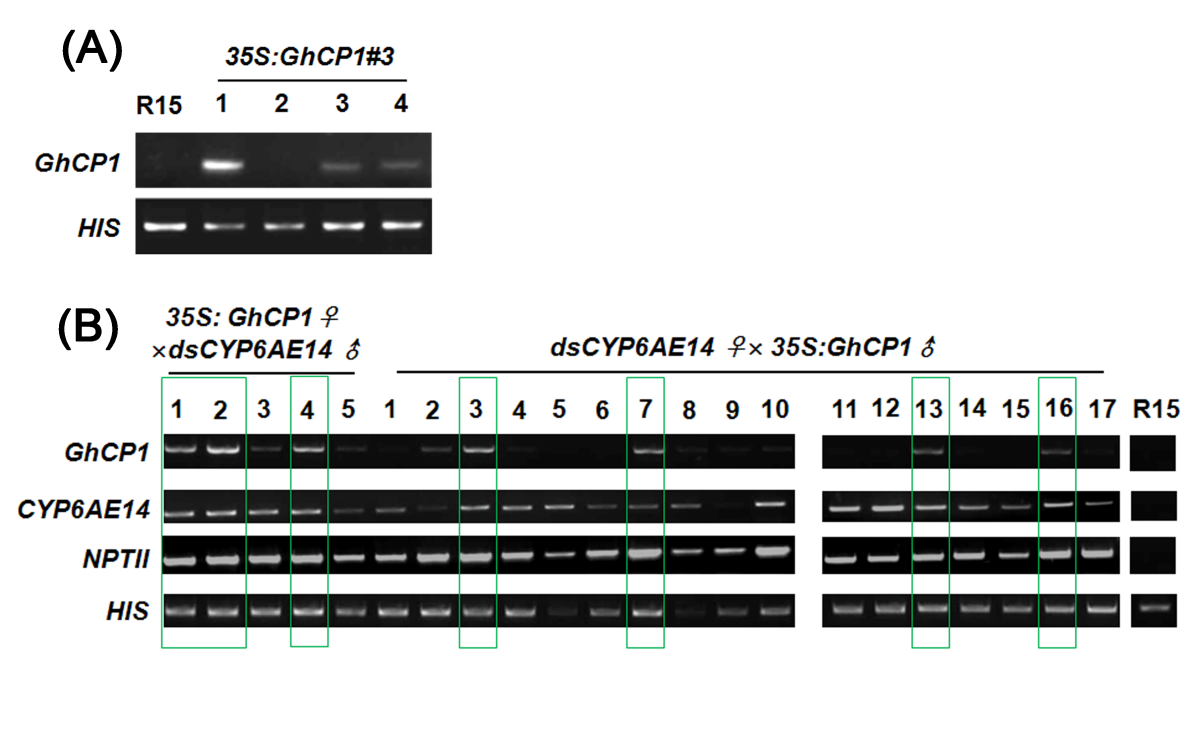


**Figure S7.** RT-PCA detection of *GhCP1* and *dsCYP6AE14* expression in *35S:GhCP1#3* and crossed cotton plants*.*

**a** *GhCP1* expressions in wild-type (R15) and T1 generation of *35S:GhCP1#3.* T1 plants of *35S:GhCP1#3-1* cotton overexpressing *GhCP1* were used for insect feeding assay. **b** *GhCP1*, *dsCYP6AE14* and *NPTII* expressions in 17 individuals of crossed (F1 generation) and wild-type (R15) cotton leaves were analyzed by RT-PCR. Green boxes indicate the transgenic lines expressing both *GhCP1* and *dsCYP6AE14*, which were used for insect feeding assay. Young leaf (second from the top) from 4-week-old cotton plants was used for analysis. Expression of *Histon 3* (*HIS*) was used as a reference.


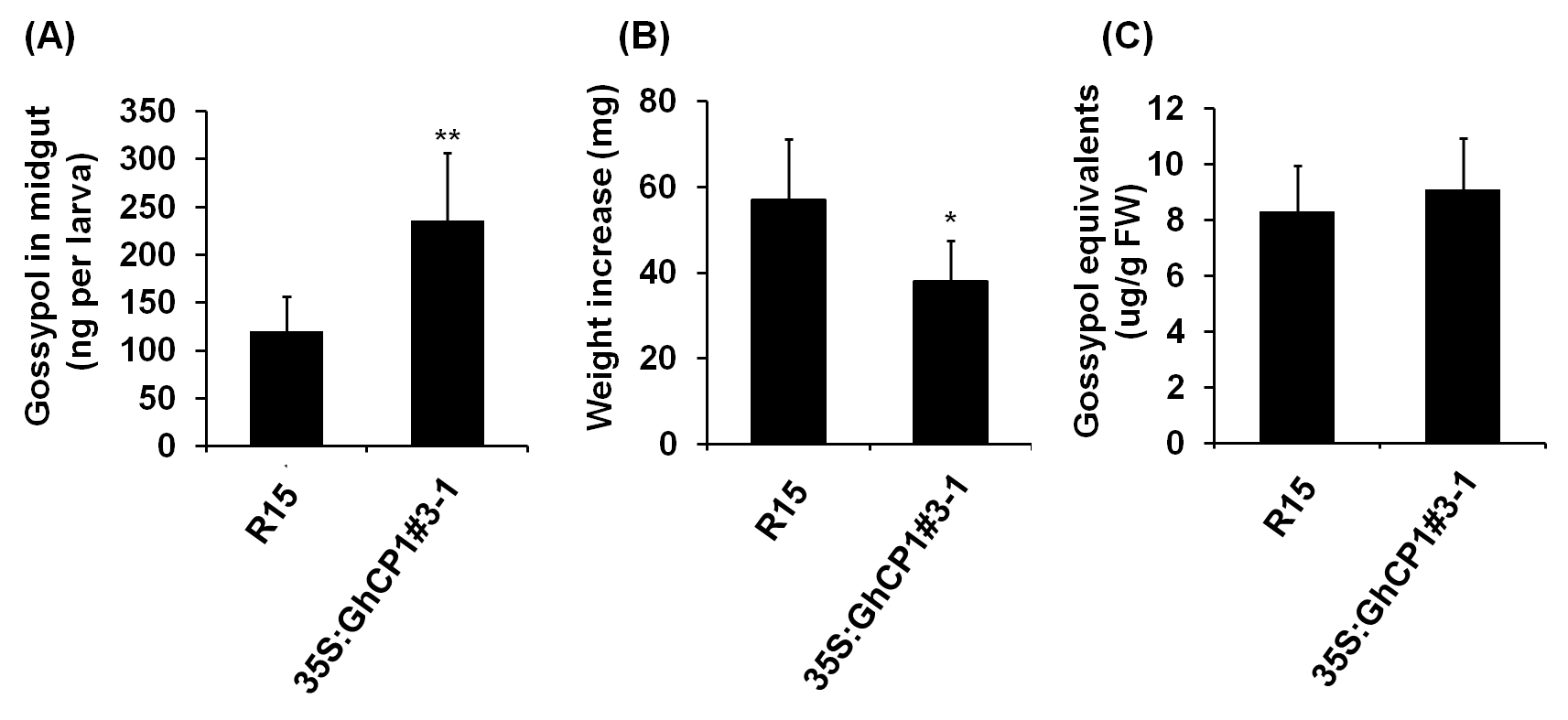


**Figure S8.** Adverse effect of *35S:GhCP1#3-1* on bollworm growth.

**a** Accumulation of gossypol in midgut. **b** Weight increase of tested larvae. **c** Level of gossypol equivalents in tested cotton plant leaves. The 3rd instar larvae were fed on leaves of wild-type (R15) and *35S:GhCP1#3-1* for 5 days. Gossypol accumulation in midgut and bollworm weight increase were recorded. *: P<0.05, **: P<0.01. Error bars represent standard deviation.
